# Integrated Transcriptomic and Machine Learning Analysis Reveals Immune-Related Regulatory Networks in Anti-NMDAR Encephalitis

**DOI:** 10.3390/ijms27021044

**Published:** 2026-01-21

**Authors:** Kechi Fang, Xinming Li, Jing Wang

**Affiliations:** 1 State Key Laboratory of Cognitive Science and Mental Health, Institute of Psychology, Chinese Academy of Sciences, Beijing 100101, China; 2 Department of Psychology, University of Chinese Academy of Sciences, Beijing 100049, China

**Keywords:** anti-NMDAR encephalitis, transcriptomic profiling, immune-related biomarkers, ceRNA regulatory network, tumor–immune–neural axis

## Abstract

Anti-N-methyl-D-aspartate receptor (anti-NMDAR) encephalitis is an immune-mediated neurological disorder driven by dysregulated neuroimmune interactions, yet the molecular architecture linking tumor-associated immune activation, peripheral immunity, and neuronal dysfunction remains insufficiently understood. In this study, we established an integrative computational framework that combines multi-tissue transcriptomic profiling, weighted gene co-expression network analysis, immune deconvolution, and machine learning-based feature prioritization to systematically characterize the regulatory landscape of the disease. Joint analysis of three independent GEO datasets spanning ovarian teratoma tissue and peripheral blood transcriptomes identified 2001 consistently dysregulated mRNAs, defining a shared tumor–immune–neural transcriptional axis. Across multiple feature selection algorithms, *ACVR2B* and *MX1* were reproducibly prioritized as immune-associated candidate genes and were consistently downregulated in anti-NMDAR encephalitis samples, showing negative correlations with neutrophil infiltration. Reconstruction of an integrated mRNA-miRNA-lncRNA regulatory network further highlighted a putative core axis (*ENSG00000262580*–hsa-miR-22-3p–*ACVR2B*), proposed as a hypothesis-generating regulatory module linking non-coding RNA regulation to immune-neuronal signaling. Pathway and immune profiling analyses demonstrated convergence of canonical immune signaling pathways, including JAK-STAT and PI3K-Akt, with neuronal communication modules, accompanied by enhanced innate immune signatures. Although limited by reliance on public datasets and small sample size, these findings delineate a systems-level neuroimmune regulatory program in anti-NMDAR encephalitis and provide a scalable, network-based multi-omics framework for investigating immune-mediated neurological and autoimmune disorders and for guiding future experimental validation.

## 1. Introduction

Anti-N-methyl-D-aspartate receptor (anti-NMDAR) encephalitis is a representative autoimmune encephalitis characterized by pathogenic autoantibodies against the GluN1 subunit of neuronal surface NMDARs. Clinically, it manifests with prominent neuropsychiatric symptoms, seizures, and cognitive deficits [1,2]. While immunotherapy alleviates symptoms in many patients, a substantial proportion experience incomplete recovery or relapse, indicating that the molecular mechanisms driving disease initiation and progression remain incompletely understood.

Immune dysregulation is central to disease pathogenesis. B-cell mediated autoimmunity drives neuroinflammation in experimental models [3], and patient studies have reported aberrant expansion of CD20+ B cells [4], altered neutrophil-to-lymphocyte ratios [5], and elevated B-cell activating factors in cerebrospinal fluid [6]. Recent transcriptomic studies of peripheral blood and cerebrospinal fluid further suggest broad immune activation and interferon-related signaling in anti-NMDAR encephalitis, supporting a role for systemic immune transcriptional reprogramming in disease progression [7,8]. However, these studies have largely focused on single tissue compartments and gene-level changes, leaving the upstream regulatory architecture coordinating immune and neuronal dysfunction insufficiently defined.

Non-coding RNAs, including microRNAs (miRNAs) and long non-coding RNAs (lncRNAs), act as key regulators of gene expression through competitive endogenous RNA (ceRNA) interactions, influencing immune signaling and neuronal pathways [9]. Dysregulated ceRNA networks have been implicated in neuroinflammatory and autoimmune diseases such as multiple sclerosis, systemic lupus erythematosus, and Parkinson’s disease [10,11,12,13]. Despite the recognized immunological complexity of anti-NMDAR encephalitis, ceRNA-mediated regulatory mechanisms have not yet been systematically explored in this disease context, representing a critical knowledge gap.

Importantly, anti-NMDAR encephalitis is uniquely associated with ovarian teratomas, which are thought to act as immune-priming sites through aberrant expression of neuronal antigens. How tumor-associated immune activation propagates into peripheral immune dysregulation and ultimately impacts neuronal function remains poorly understood at a systems level. An integrative, multi-tissue analytical strategy is therefore required to capture this tumor–immune–neural continuum.

In this study, we integrated multi-omics transcriptomic datasets from peripheral blood and disease-associated ovarian teratomas to profile mRNA, miRNA, and lncRNA expression. By combining weighted gene co-expression network analysis (WGCNA), functional enrichment, immune cell deconvolution, and machine learning-based feature prioritization, we constructed a multi-layer regulatory network that moves beyond single-gene analysis toward network-level inference. This approach identifies immune-associated hub genes and generates testable hypotheses regarding regulatory interactions underlying anti-NMDAR encephalitis, providing systems-level insight into disease-associated neuroimmune dysregulation.

## 2. Results

### 2.1. Dysregulated Transcriptomic Profiles in Anti-NMDAR Encephalitis

**Figure 1:**
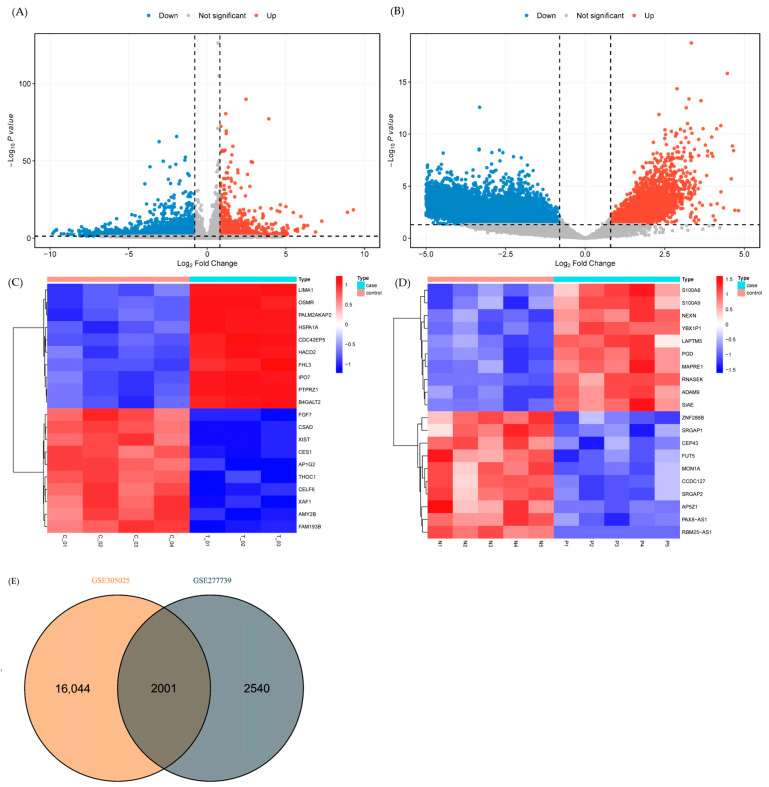
Differential expression analysis of mRNAs in anti-NMDAR encephalitis. (**A**) Volcano plot of differentially expressed mRNAs in ovarian teratoma tissue, showing 4541 dysregulated genes (1295 upregulated and 3246 downregulated). (**B**) Volcano plot of differentially expressed mRNAs in peripheral blood samples, including 18,045 dysregulated mRNAs (2600 upregulated and 15,445 downregulated). (**C**,**D**) Heatmaps depicting the top 25 up- and downregulated differentially expressed mRNAs ranked by log_2_ fold change in ovarian teratoma tissue (**C**) and peripheral blood (**D**), highlighting prominent tissue-specific and systemic transcriptional alterations. (**E**) Venn diagram displaying the intersection of differentially expressed mRNAs between tissue and blood datasets, revealing 2001 genes consistently dysregulated across sample types.

Transcriptomic profiling revealed extensive dysregulation of coding and non-coding RNAs in anti-NMDAR encephalitis, encompassing tissue-specific and systemic components. Details of the datasets included in this study were provided in Table 1. In ovarian teratoma tissue associated with anti-NMDAR encephalitis, 4541 mRNAs were differentially expressed, including 1295 upregulated and 3246 downregulated genes (Figure 1A). Peripheral blood analysis identified 18,045 differentially expressed mRNAs (2600 upregulated, 15,445 downregulated; Figure 1B) and 2309 lncRNAs (79 upregulated, 2230 downregulated), as well as 28 miRNAs (16 upregulated, 12 downregulated) (Figure S1).

To highlight the most prominently dysregulated genes, heatmaps were generated depicting the top 25 up- and downregulated mRNAs ranked by log_2_ fold change in ovarian teratoma tissue and peripheral blood (Figure 1C,D). The volcano plots and heatmaps of differential expression results for mRNAs were shown in Figure 1A–D.

Intersection analysis across tissue and blood datasets identified 2001 mRNAs consistently dysregulated, representing a core transcriptional signature of anti-NMDAR encephalitis (Figure 1E). The convergence of tissue and blood expression profiles supports a model in which antigen-driven immune activation in ovarian teratomas elicits systemic transcriptional alterations detectable in peripheral blood.

### 2.2. Weighted Gene Co-Expression Network Analysis Reveals Disease-Associated Modules

**Figure 2:**
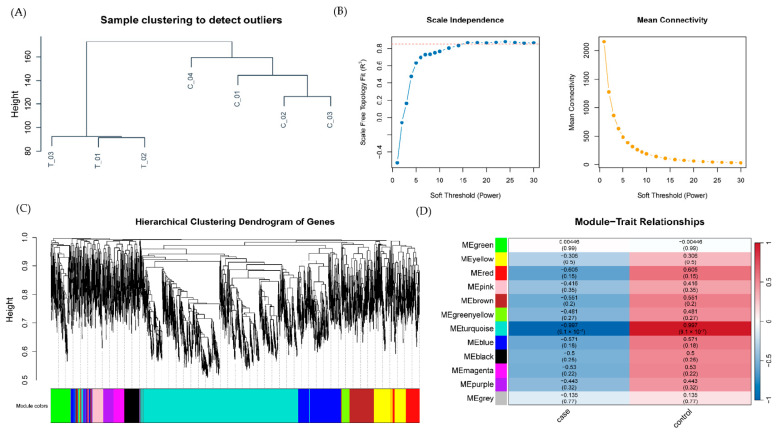
Weighted gene co-expression network analysis (WGCNA). (**A**) Sample clustering dendrogram; each branch represents a sample and no outliers were detected. (**B**) Determination of optimal soft-thresholding power (β) to ensure scale-free topology. (**C**) Gene module identification and merging under hierarchical clustering dendrogram. (**D**) Heatmap of correlations between module eigengenes and clinical traits, highlighting the turquoise module as strongly associated with anti-NMDAR encephalitis.

To investigate coordinated transcriptional changes, weighted gene co-expression network analysis (WGCNA) was performed on mRNA expression profiles. Sample clustering demonstrated no significant outliers, confirming high-quality data for network construction (Figure 2A). A soft-thresholding power of β = 16 was selected to achieve scale-free topology (R^2^ > 0.85; Figure 2B). Hierarchical clustering with dynamic tree cutting identified 12 distinct gene modules (Figure 2C).

**Figure 3:**
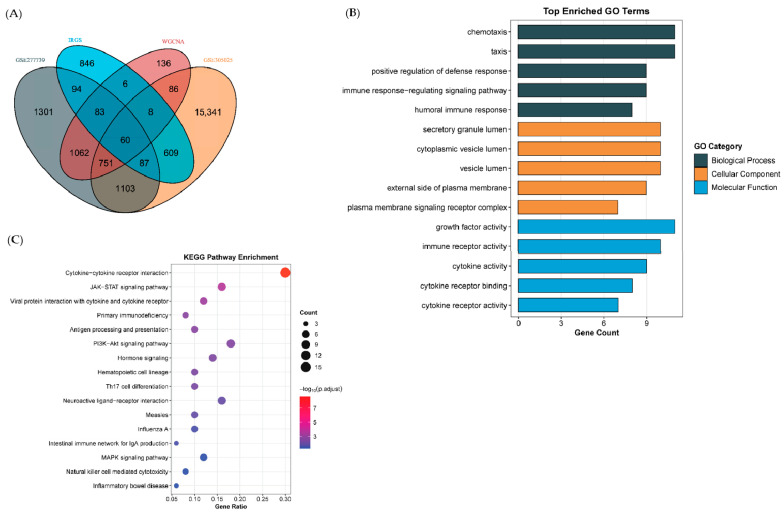
Identification and functional enrichment of immune-related hub genes. (**A**) Venn diagram showing the intersection of differentially expressed mRNAs, turquoise module genes, and ImmPort immune-related genes (60 overlapping genes). (**B**) KEGG pathway enrichment highlighting immune activation, inflammatory signaling, and neural-related pathways. (**C**) GO enrichment analysis across biological processes (BP), cellular components (CC), and molecular functions (MF).

Module-trait correlation analysis revealed that the turquoise module exhibited the strongest negative association with anti-NMDAR encephalitis, suggesting its genes are tightly linked to disease pathophysiology (Figure 2D). This module comprised 2192 genes, which were further intersected with the previously identified differentially expressed mRNAs and 1793 immune-related genes from the ImmPort database to isolate 60 immune-related hub genes (Figure 3A, Table S1). These hub genes represent key candidates mediating immune and neuronal dysregulation in anti-NMDAR encephalitis.

### 2.3. Functional Enrichment Analysis Highlights Immune and Neural Pathways

Gene Ontology (GO) and Kyoto Encyclopedia of Genes and Genomes (KEGG) enrichment analyses were performed to elucidate the biological roles of the 60 immune-related hub genes. KEGG pathways were enriched in immune activation and inflammation-related pathways, including cytokine–cytokine receptor interaction, JAK-STAT, MAPK, and PI3K-Akt pathways, as well as antigen processing and presentation, primary immunodeficiency, Th17 cell differentiation, and neuroactive ligand–receptor interaction (Figure 3B, Tables S2 and S3).

GO analysis revealed that hub genes predominantly participate in chemotaxis, humoral immune response, and immune response-regulating signaling (biological processes, BP); localize to secretory granule lumen, cytoplasmic vesicle lumen, and vesicle lumen (cellular components, CC); and possess molecular functions such as growth factor activity, immune receptor activity, cytokine receptor activity, gonadotropin hormone-releasing hormone activity, and nerve growth factor receptor binding (Figure 3C). These findings suggest that the hub genes orchestrate immune-neuronal interactions and inflammatory responses central to disease progression.

### 2.4. mRNA-miRNA-lncRNA Regulatory Network Analysis

**Figure 4:**
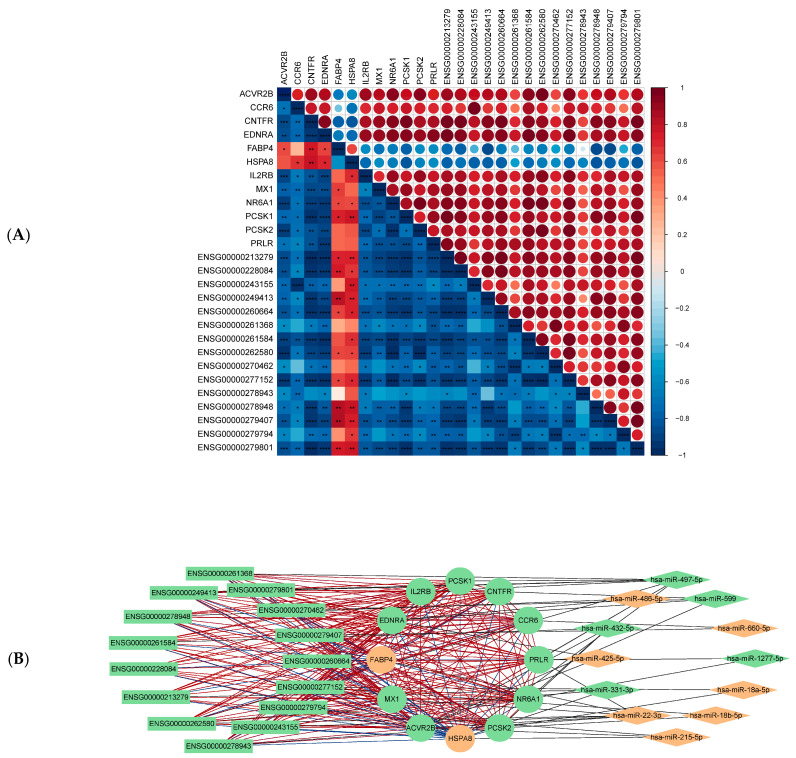
mRNA-miRNA-lncRNA regulatory network. (**A**) Pearson correlation heatmap of mRNA and lncRNA expression profiles. The upper triangle shows correlation coefficients, with color intensity and circle size reflecting correlation strength, while the lower triangle shows corresponding *p*-values (* *p* ≤ 0.05; ** *p* ≤ 0.01; *** *p* ≤ 0.001; **** *p* ≤ 0.0001). (**B**) Integrated mRNA-miRNA-lncRNA regulatory network. lncRNAs are labeled using standardized GENECODE/Ensembl gene identifiers (see Supplementary Table S4). Node shapes denote RNA type (circles, mRNAs; diamonds, miRNAs; squares, lncRNAs), and node colors indicate differential expression status (orange, upregulated; green, downregulated). Black edges indicate validated or predicted miRNA-target interactions, whereas colored edges denote mRNA-lncRNA co-expression relationships (red, positive correlation; blue, negative correlation; color intensity reflects correlation strength).

Using the ENCORI/starBase database, target prediction for 28 differentially expressed miRNAs identified 22,719 miRNA-mRNA and 418 miRNA-lncRNA pairs. Integration with the 60 immune-related hub genes and 2309 differentially expressed lncRNAs refined the network to 27 miRNA-mRNA and 19 miRNA-lncRNA interactions. Pearson correlation analysis of mRNA and lncRNA profiles yielded 126 significant mRNA-lncRNA pairs (|r| > 0.8, *p* < 0.05; Figure 4A, Table S4).

The final integrated mRNA-miRNA-lncRNA regulatory network comprised 39 nodes (12 mRNAs, 12 miRNAs, 15 lncRNAs) and 172 edges (Figure 4B, Table S4). Network topology analysis revealed a core regulatory axis: *ACVR2B* (downregulated, degree = 21)—hsa-miR-22-3p (upregulated, degree = 7)—*ENSG00000262580* (downregulated, degree = 12). This axis follows the classic ceRNA model, where reduced *ENSG00000262580* may enhance miR-22-3p-mediated suppression of *ACVR2B*, potentially diminishing neuroprotection promoting immune imbalance.

### 2.5. Machine Learning-Prioritized Features

To prioritize candidate genes within the inferred regulatory network, three complementary machine learning algorithms (LASSO, SVM, and random forest) were applied to the batch-corrected dataset. Given the limited sample size, these algorithms were used exclusively for feature prioritization rather than predictive modeling.

**Figure 5:**
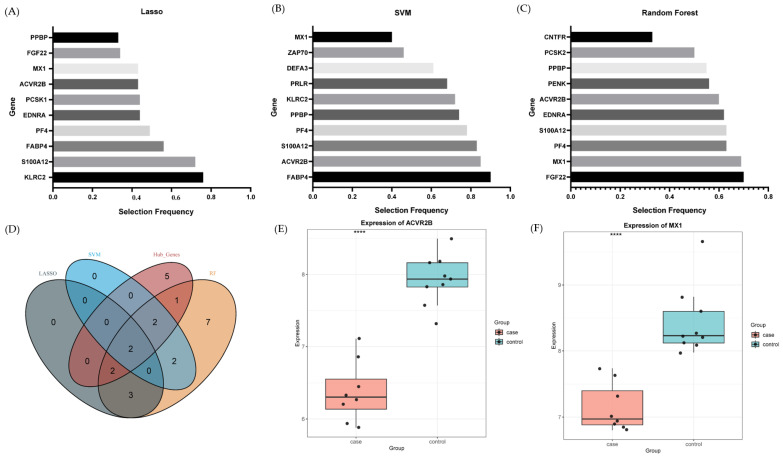
Machine learning-based prioritization of candidate genes. (**A**) Genes selected by LASSO regression across repeated subsampling iterations in the batch-corrected integrated dataset. (**B**) Genes selected by linear support vector machine (SVM) based on absolute feature weights across repeated subsampling iterations. (**C**) Genes prioritized by random forest according to MeanDecreaseGini importance across repeated subsampling iterations. (**D**) Venn diagram showing the intersection of candidate genes identified by LASSO, SVM, and random forest, yielding two consensus genes, *ACVR2B* and *MX1*, which were also present in the mRNA-miRNA-lncRNA regulatory network. (**E**,**F**) Box-and-jitter plots showing expression levels of *ACVR2B* and *MX1* in anti-NMDAR encephalitis and control samples across the integrated dataset. Group difference were assessed using Mann–Wilcoxon U tests (**** *p* < 0.0001).

Across repeated subsampling iterations, LASSO, SVM and RF each identified a set of recurrently selected genes (Figure 5A–C). Intersection of the three algorithm-specific candidate sets and subsequent overlap with the mRNA-miRNA-lncRNA regulatory network yielded two consensus genes, ACVR2B and MX1 (Figure 5D; Supplementary Table S5).

Expression analysis showed that both *ACVR2B* and *MX1* were significantly downregulated in anti-NMDAR encephalitis samples compared with controls in both ovarian teratoma tissues and peripheral blood datasets (Figure 5E). Although nominal *p*-values were used during initial DEG screening, both genes remained significantly downregulated after multiple-testing correction in each independent dataset (Supplementary Table S6), supporting the robustness of their differential expression.

### 2.6. Immune Cell Infiltration Analysis Uncovers Microenvironmental Alterations

**Figure 6:**
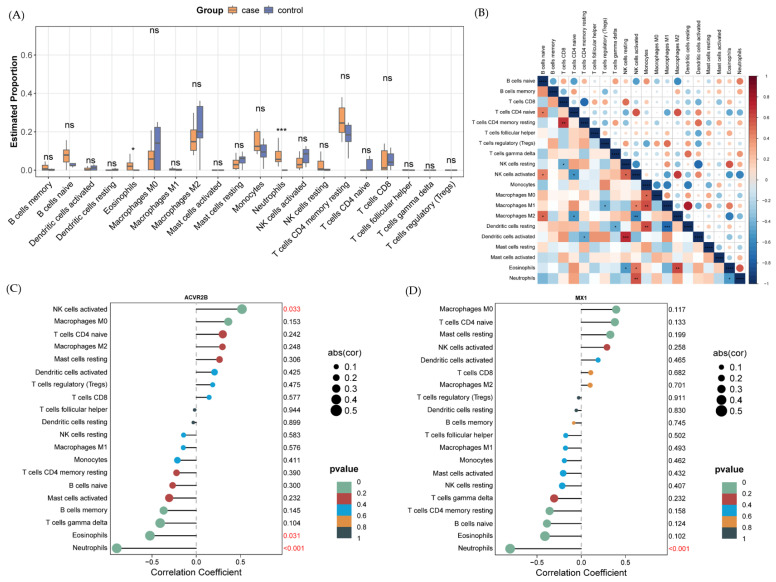
Immune cell infiltration and correlations. (**A**) Box plots comparing immune cell abundances between control and anti-NMDAR encephalitis samples (* *p* ≤ 0.05; *** *p* ≤ 0.001). ns denotes not significant. (**B**) Correlation heatmap of immune cell types. Upper triangle shows correlation coefficients, lower triangle represents *p*-values (* *p* ≤ 0.05; ** *p* ≤ 0.01; *** *p* ≤ 0.001, **** *p* ≤ 0.0001). (**C**,**D**) Lollipop and scatter plots illustrating correlations between *ACVR2B* (C) and *MX1* (D) expression and immune cell abundances. Circle size indicates absolute correlation, color represents *p*-value, R denotes Spearman correlation coefficient.

CIBERSORT analysis estimated the relative abundances of 20 immune cell types, revealing elevated neutrophils and eosinophils in anti-NMDAR encephalitis samples (*p* < 0.05; Figure 6A). Correlation analysis identified a strong positive relationship between neutrophils and eosinophils (r > 0.6; Figure 6B), suggesting cooperative roles in disease-associated inflammation.

Spearman correlation demonstrated that *ACVR2B* and *MX1* expression negatively correlated with neutrophils abundance (Figure 6C,D, respectively), implying that these genes may normally suppress neutrophil activation. The downregulation of these genes may lead to excessive activation of neutrophils, exacerbating inflammatory responses, linking transcriptional dysregulation to immune-mediated neuronal damage.

## 3. Discussion

This study presents a systems-level computational investigation of anti-NMDAR encephalitis through the integrative analysis of transcriptomic data derived from ovarian teratoma tissue and peripheral blood. By combining multi-compartment transcriptomics, weighted gene co-expression network analysis, immune cell deconvolution, and machine learning-based feature prioritization, we aimed to construct a hierarchical regulatory network linking tumor-associated immune activation to systemic neuroimmune dysregulation. Importantly, this work is intended as a hypothesis-generating study and provides a framework for guiding subsequent experimental validation rather than establishing definitive causal relationships.

Three main contributions can be highlighted. First, we established a shared tumor–immune–neural transcriptional network cross tissue compartments. Second, we identified key immune-associated features via convergence of multiple machine learning algorithms rather than single-method prioritization. Third, we constructed a multi-layer mRNA-miRNA-lncRNA regulatory network providing mechanistic insight into post-transcriptional regulation in a neuroimmune context.

Previous studies of anti-NMDAR encephalitis predominantly focused on cerebrospinal fluid immunology or peripheral immune phenotyping, including antibody profiles and immune cell composition [14], with few addressing ovarian teratoma as an immunogenic trigger in paraneoplastic cases [15,16]. Systematic transcriptomic comparisons between tumor tissue and peripheral blood have not been reported previously. Our analysis identified 2001 mRNAs consistently dysregulated across both tissue types, suggesting a shared transcriptional program. This supports a model in which local tumor-associated immune perturbations may propagate through immune signaling networks to induce systemic immune reprogramming [17].

Functional enrichment of immune-related hub genes revealed convergence of canonical pathways, including cytokine–cytokine receptor interaction, JAK-STAT, and PI3K-Akt, with neuronal signaling modules. This dual enrichment indicates that immune activation and neuronal regulation are closely intertwined For example, JAK-STAT signaling mediate neuroinflammation in astrocytes and microglia [18,19], while PI3K-Akt contributes to B-cell differentiation and antibody production [20,21]. Notably, canonical inflammasome-related pathways, such as NLRP3 signaling, were not strongly enriched in our intersected dataset. This suggests that in the context of anti-NMDAR encephalitis, innate immune activation may be primarily cytokine-driven.

Machine learning-based prioritization consistently highlighted *ACVR2B* and *MX1* as central immune-associated features, with concordant downregulation observed in both ovarian teratoma tissue and peripheral blood. Although neither gene has been directly implicated in anti-NMDAR encephalitis previously, existing research in related neuroinflammatory contexts provides a compelling biological basis for their relevance.

*MX1* (Myxovirus resistance protein 1) is a key effector of the type I interferon (IFN) system [22]. Unlike multiple sclerosis, where *MX1* is significantly upregulated in brain lesions as a marker of active IFN-β response [23,24], the downregulation observed here suggests a distinct immunological signature. Given that viral prodromes frequently precede anti-NMDAR encephalitis [25], the diminished *MX1* expression may reflect a state of immune exhaustion or a blunted interferon-mediated defense, potentially facilitating the transition from an initial antiviral response to systemic autoimmunity.

Concurrently, *ACVR2B*, a type IIB receptor for activin signaling, functions as a key regulator of the oligodendrocyte lineage and myelin repair [26]. Research indicates that *ACVR2B* signaling is indispensable for the differentiation of progenitor cells into mature myelin-producing cells following inflammatory injury [27,28,29]. The consistent downregulation of *ACVR2B* in our cohort suggests a compromised innate immune gatekeeping (low *MX1*) paired with a failure of endogenous neurorepair signaling (low *ACVR2B*). This dual vulnerability may explain the persistent neurological deficits observed in patients despite immunotherapy.

The multi-layer mRNA-miRNA-lncRNA regulatory network provided additional mechanistic resolution. The proposed *ENSG00000262580*-hsa-miR-22-3p-*ACVR2B* axis illustrated how non-coding RNAs may fine-tune immune-neuronal signaling at the post-transcriptional level. miR-22-3p regulates NF-κB activation and cytokine expression [30,31], suggesting that altered upstream lncRNA regulation could influence *ACVR2B* expression and downstream inflammatory signaling. This ceRNA axis should be regarded as a hypothesis-generating regulatory, serving as a prioritization of candidate regulatory interactions rather than a confirmed mechanistic pathway.

Immune deconvolution analysis revealed elevated neutrophil and eosinophil signatures with a strong positive correlation between these innate immune populations. Neutrophils have increasingly been implicated autoimmune amplification through mechanisms such as extracellular trap formation and antigen exposure [32,33]. The negative correlation between *ACVR2B/MX1* expression and neutrophil abundance suggest that the reduced expression of these regulatory features may permit exaggerated innate immune activation. Together, these observations provide a systems-level framework for understanding how tumor-driven immune perturbations could extend into peripheral immune dysregulation and ultimately contribute to neuroimmune pathology.

Several limitations should be acknowledged. First, this study relied on public transcriptomic datasets with small sample sizes. Although cross-compartment integration was employed to improve robustness, the results should be interpreted cautiously due to potential batch effect. Accordingly, machine learning was utilized strictly for feature prioritization rather than predictive modeling. Second, while enrichment analyses incorporated multiple-testing adjustment, initial differential expression screening used nominal *p*-values to preserve shared signals across limited cohorts. Importantly, we confirmed that our top prioritized targets (*ACVR2B* and *MX1*) remain statistically significant even after multiple-testing correction (Adjusted *p* < 0.05) across datasets.

To transition these findings from in silico prediction to biological verification, we propose a comprehensive validation framework (Figure S2). Future experimental efforts will include: verifying the downregulation of *ACVR2B*, *MX1* via quantitative PCR in independent patient cohorts; corroborating the inferred immune cell dynamics using flow cytometry-based immunophenotyping; and elucidating the mechanistic impact of these regulatory axes on neuroimmune crosstalk through targeted functional assays in cell-based models.

## 4. Materials and Methods

### 4.1. Data Acquistion and Preprocessing

Transcriptomic datasets for anti-NMDAR encephalitis were retrieved from the Gene Expression Omnibus (GEO) database “https://www.ncbi.nlm.nih.gov/geo/ (accessed on 10 October 2025)” (Table 1). Three complementary datasets were selected to capture multi-level transcriptional dysregulation. GSE277739 (mRNA sequencing from 3 anti-NMDAR encephalitis-associated ovarian teratomas versus 4 control tissue samples), GSE305025 (mRNA and lncRNA sequencing from peripheral blood of 5 patients versus 5 healthy controls), and GSE287388 (miRNA sequencing from peripheral blood of 9 patients versus 8 healthy controls).

Raw count matrices were downloaded and processed using a standardized workflow. Gene identifiers were unified by mapping Entrez IDs to Ensembl gene symbols using GENCODE v49 reference annotations (gencode.v49.annotation.gtf; Ensembl release 115) to ensure consistency across datasets.

Non-coding RNAs were annotated according to GENCODE reference annotations (Ensembl release 115) [34], which provide comprehensive and curated gene models for human coding and non-coding transcripts. Long non-coding RNAs were uniformly referred to as lncRNAs throughout the manuscript to ensure terminological consistency.

Immune-related gene sets (IRGS) were obtained from the ImmPort database “https://www.immport.org/home (accessed on 15 October 2025)”, a curated repository of genes broadly involved in immune processes, including cytokine signaling, innate and adaptive immune responses, and immune cell activation [35].

As all data were acquired from publicly available repositories, no additional institutional ethics approval was required.

### 4.2. Differential Expression Analysis

Differential expression analysis was independently performed for each dataset using DESeq2 R package (v1.38.3) [36]. Genes with low mean abundance (mean count < 1 across all samples) were excluded to reduce noise and improve statistical power. Differentially expressed genes were defined using a nominal *p*-value < 0.05 and |log_2_ fold change (FC)| > 0.8.

Given the extremely small sample sizes of the individual datasets, multiple testing correction at this initial DEG screening stage resulted in very few significant genes and minimal overlap across datasets. Therefore, nominal *p*-values were used for DEG identification as an exploratory feature selection step, followed by cross-dataset intersection and network-based filtering to enhance robustness. This DEG analysis should be regarded as hypothesis-generating rather than confirmatory.

Volcano plots were generated using the ggplot2 R package (v3.5.2) [37], and heatmaps displaying the top 25 up- and downregulated genes ranked by |log_2_ FC| were constructed using the pheatmap R package (v1.0.13). Overlapping DEGs across datasets was visualized using the VennDiagram R package (v1.7.3).

### 4.3. Weighted Gene Co-Expression Network Analysis (WGCNA)

To identify disease-relevant co-expression modules beyond pairwise comparisons, WGCNA (v1.73) [38] was applied to the GSE277739 dataset.

Genes exhibiting high variability (top 5000 ranked by median absolute deviation, MAD) were selected to focus on biologically informative features. Genes with missing values or zero variance were excluded. The soft-thresholding power (β) was determined using the pickSoftThreshold function, selecting the lowest β achieving scale-free topology (R^2^ ≥ 0.85) while maintaining adequate mean connectivity.

This matrix was transformed into a topological overlap matrix (TOM) to measure network interconnectedness. Genes were clustered based on the TOM distance using average linkage, and modules were defined by dynamic tree cutting (minModuleSize = 75, mergeCutHeight = 0.25). Module eigengenes (MEs) were correlated (Pearson’s r) with clinical traits (patient vs. control). Modules with |r| > 0.8 and *p* < 0.05 were considered disease-associated and prioritized for downstream analysis.

### 4.4. Identification of Immune-Related Hub Genes and Functional Enrichment Analysis

Genes within disease-associated WGCNA-modules were intersected with DEGs identified independently from GSE277739 and GSE305025 to obtain candidate genes consistently dysregulated across tumor and peripheral blood compartments. These genes were further intersected with ImmPort immune-related genes to define immune-related hub genes. ImmPort genes were used as a broad immune annotation reference rather than being specific to anti-NMDAR encephalitis.

Functional characterization of these key genes was conducted using clusterProfiler R package (v3.5.2) [39] for Gene Ontology (GO) and Kyoto Encyclopedia of Genes and Genomes (KEGG) enrichment analyses. For all enrichment analysis, multiple testing correction was applied, and GO terms and KEGG pathways with adjusted *p* < 0.05 and gene count > 1 were considered statistically significant. Results were visualized using ggplot2 R package (v3.5.2) [37].

### 4.5. Construction of the mRNA-miRNA-lncRNA Regulatory Network

To explore potential competing endogenous RNA (ceRNA) regulatory mechanisms involving immune-related hub genes, an integrated mRNA-miRNA-lncRNA network was constructed.

Experimentally validated and predicted miRNA–mRNA and miRNA–lncRNA interactions were retrieved from ENCORI/starBase (https://rnasysu.com/encori) (accessed on 15 October 2025) [40]. Only miRNAs predicted to target immune-related hub genes were retained.

mRNA-lncRNA co-expression relationships were evaluated using Pearson correlation based on expression profiles from GSE305025, implemented via the rcorr function in the Hmisc package (v5.2.3). Significant co-expression was defined as |r| > 0.8 and *p* < 0.05, representing potential cis- or trans-regulatory interactions.

The integrated regulatory network was assembled and visualized using Cytoscape (v 3.10.4) [41], with node size indicating degree centrality and edge color denoting interaction type. lncRNAs were labeled using gene symbols rather than Ensemble sequence ID to improve interpretability.

### 4.6. Machine Learning-Based Feature Selection

To prioritize robust candidate features among immune-related hub genes, transcriptomic data from GSE277739 and GSE305025 were merged, and potential batch effects were corrected using the ComBat algorithm implemented in the sva R package. The resulting integrated dataset comprised eight anti-NMDAR encephalitis samples and nine control samples.

Given the limited cohort size, machine learning algorithms were applied exclusively as feature screening tools rather than for predictive model construction, in order to reduce overfitting and improve robustness. Three complementary algorithms, including least absolute shrinkage and selector operation (LASSO) [42], linear support vector machine (SVM) [43], and random forest (RF) [44], were employed to identify convergent features under repeated subsampling.

A stability-selection method was implemented with 100 rounds of repeated stratified subsampling. In each iteration, six patient samples and six control samples were randomly selected to form a balanced training set, providing a compromise between class balance and variance control under small-sample conditions. Within each iteration, LASSO regression (α = 1) was implemented using the glmnet package (v4.1.10) [45] and genes with non-zero coefficients at the optimal regularization parameter determined by internal cross-validation were retained. Random forest models were constructed with 500 trees using the randomForest R package (v4.7.1.2), and the top 10 genes ranked by MeanDecreaseGini importance were retained. Linear SVM models (cost = 1) were implemented using the e1071 package, and the top 10 genes ranked by the absolute magnitude of feature weights were selected.

For each algorithm, the union of all genes appearing in the top-ranked feature sets across the 100 iterations was defined as its candidate feature pool. To further enhance biological plausibility and reduce false-positive discoveries, the intersection of the candidate feature pools from all three algorithms was subsequently overlapped with the pre-established mRNA–miRNA–lncRNA regulatory network. Genes supported by both multi-algorithm stability and network-level regulatory context were prioritized as final candidate features.

Expression differences between anti-NMDAR encephalitis and control groups were assessed using the Mann–Whitney U test (*p* < 0.05), and expression distributions were visualized using boxplots generated with the ggplot2 package.

### 4.7. Immune Cell Infiltration Analysis

To characterize the immune microenvironment associated with anti-NMDAR encephalitis, immune cell composition was inferred using CIBERSORT (Cell-type Identification By Estimating Relative Subsets Of RNA Transcripts) [46] with LM22 leukocyte gene signature matrix. The analysis was performed on the merged, batch-corrected dataset with 1000 permutations.

Differences in immune cell proportions between patient and control were evaluated using Wilcoxon rank-sum tests (*p* < 0.05). Spearman’s correlation analysis was performed to evaluate associations between immune cell abundances and candidate gene. Results were visualized using ggplot2 as boxplots, correlation heatmaps, and lollipop charts.

## 5. Conclusions

In summary, this study presents a multi-layered, systems-level computational framework for investigating tumor-associated neuroimmune regulation in anti-NMDAR encephalitis. By integrating multi-tissue transcriptomics, co-expression network analysis, immune deconvolution, and machine learning-based feature prioritization, we identified a shared tumor–immune–neural transcriptional program and highlighted *ACVR2B* and *MX1* as key immune-associated features consistently downregulated in anti-NMDAR encephalitis samples. We further proposed a putative ceRNA regulatory axis involving *ENSG00000262580*–hsa-miR-22-3p–*ACVR2B*.

Given the reliance on publicly available datasets and limited sample sizes, these findings should be regarded as hypothesis-generating rather than definitive. Nevertheless, the network-oriented regulatory model provides mechanistic insights into how tumor-associated immune perturbations may propagate into systemic neuroimmune dysregulation. This integrative analytic strategy is broadly applicable to other immune-mediated neurological and autoimmune disorders and offers a scalable framework for future systems-level investigation.

## Figures and Tables

**Table 1:** Overview of transcriptomic datasets included in the anti-NMDAR encephalitis study.

Dataset ID	Sample Type	Disease	RNA Type	Sample Composition
GSE277739	Ovarian teratoma tissue	Anti-NMDAR encephalitis	mRNA	3 anti-NMDAR encephalitis associated teratoma, 4 non-anti-NMDAR encephalitis teratomas
GSE305025	Peripheral blood	Anti-NMDAR encephalitis	mRNA, lncRNA	5 anti-NMDAR encephalitis patients, 5 healthy controls
GSE287388	Peripheral blood	Anti-NMDAR encephalitis	miRNA	9 anti-NMDAR encephalitis patients, 8 healthy controls

## Data Availability

The raw sequence data reported in this paper have been deposited in the GEO (GSE277739, GSE305025, and GSE287388).

## Supplementary Materials

The following supporting information can be downloaded at https://www.mdpi.com/article/10.3390/ijms27021044/s1.

## Abbreviations

The following abbreviations are used in this manuscript:

ACVR2B	Activin A receptor type 2B
Anti-NMDAR	Anti-N-methyl-D-aspartate receptor
CIBERSORT	Cell-type Identification By Estimating Relative Subsets Of RNA Transcripts
ceRNA	Competitive Endogenous RNA
DEG	Differentially Expressed Genes
FC	Fold Change
FDR	False Discovery Rate
GEO	Geno Ontology
JAK-STAT	Janus Kinase–Signal Transducer and Activator of Transcription
KEGG	Kyoto Encyclopedia of Genes and Genomes
lncRNA	Long non-coding RNA
miRNA	MicroRNA
MX1	Myxovirus resistance protein 1
PI3K-Akt	Phosphoinositide 3-kinase–protein kinase B signaling pathway
WGCNA	Weighted Gene Co-expression Network Analysis
