# Supplementary figures and images for: Integrated Transcriptomic and Machine Learning Analysis Reveals Immune-Related Regulatory Networks in Anti-NMDAR Encephalitis
Source: Int J Mol Sci. 2026 Jan 21;27(2):1044. doi: 10.3390/ijms27021044 (PMC12842026; doi:10.3390/ijms27021044)

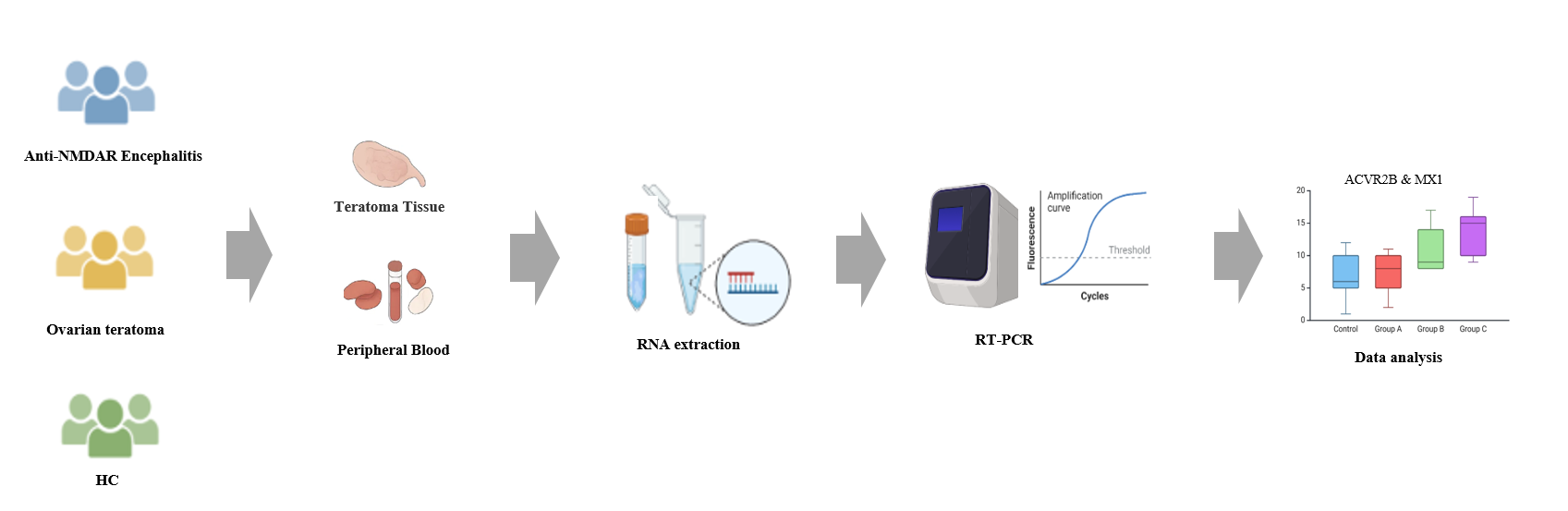

Supplement: Supplementary file 1 [file ijms-27-01044-s001.zip › Figure S2-validation.png]

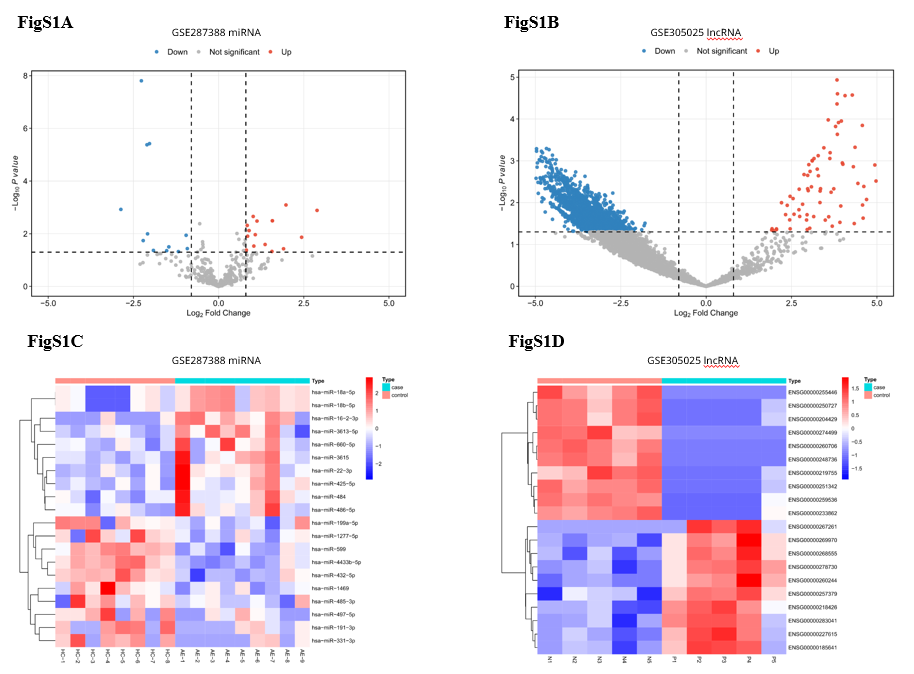

Supplement: Supplementary file 1 [file ijms-27-01044-s001.zip › FigureS1.png]
